# Perspective: Is a Closer Interaction between Experimental and Clinical Research Paradigms in Chronic Neurodegeneration, Such as Parkinson’s Disease, Necessary Again?

**DOI:** 10.3390/cells12010157

**Published:** 2022-12-30

**Authors:** Thomas Müller

**Affiliations:** Department of Neurology, St. Joseph Hospital Berlin-Weissensee, Gartenstr. 1, 13088 Berlin, Germany; th.mueller@alexianer.de or thomas.mueller@ruhr-uni-bochum.de; Tel.: +49-30-92790223; Fax: +49-30-92790703

This editorial discusses the current standstill in research in Parkinson’s disease from a clinician’s point of view. Possible causes for the failed translation of promising experimental results into clinical practice may at least partially be due to weak interaction between experimental and clinical research. Better relations between both research paradigms reflect the motivation behind this Special Issue on the molecular and cellular basis for Parkinson’s disease 2019. Its included publications predominantly result from a closer cooperation between clinical and basic researchers. This will contribute to a better understanding of Parkinson’s disease in future, which is a fascinating but still not well understood disease entity.

On the one hand, basic research on Parkinson’s disease (PD) contributed to enormous progress in the understanding of dysfunction in neuronal dopamine-synthesizing neurons and their supporting glial cells. On the other hand, the performance and publication of clinical research has become increasingly complex due an increase in bureaucratic hurdles and ethical concerns by considered carriers [1]. The translation of promising experimental outcomes with high expectations into the clinic failed with corresponding huge financial disappointments [2,3]. Moreover, clinical testing with a demand for “healthy” study participants without severe co-morbidities in pivotal trials, i.e., of a new PD drug, reflects the clinical practice only to a very limited extent [4]. In real life, PD patients always ask for a cure and/or beneficial disease modification. They are often prepared to accept the risk of side effects with new therapies if they provide hope for amelioration of PD severity or modification of progression [4]. In contrast, health authorities in cooperation with the media have become more concerned with potential side effects. They do not consider patients’ and their caregivers wishes, based on an increasing knowledge of therapy, which results from the free availability of information on the internet. Nowadays, the PD patient community is able to differentiate between promising experimental research outcomes, resulting from neuronal cell death—or fruit fly experiments, from real life outcomes in patients in relation to the financial preconditions provided by the various healthcare systems worldwide [5,6]. Moreover, PD research should focus on the real, heterogeneous nature of PD again [5]. It may be misleading to ask for the identification and description of genetically determined, underlying disease mechanisms, which may even be aggravated by PD drugs [7]. Clinicians point out that PD is not a disease. It is a disease entity [8]. The heterogeneous forms of PD, observed in the clinic, also reflect the probably multi factorial disease origins. It was and is a wrong claim that the development of PD in the whole body and not only in the brain follows a certain pattern from the clinical point of view [9,10]. Moreover it is far from clear whether neuropathological findings in PD are a consequence of the disease process only or represent the etiology of the disease [11].

A further typical example is the concept of cell- and animal models of PD, both of which predominantly focus on the central, particularly nigrostriatal cell death of dopamine synthesizing neurons associated with a deterioration of motor behaviour. However, clinicians emphasize the importance of non-motor symptoms in PD [10]. Their onset also reflects an impairment of a neurotransmission system, such as norepinephrine, in PD [10]. In the future, the idea that neuropathological abnormalities may result from the physiologic defence mechanisms against the disease process should be considered, i.e., Lewy body accumulation with misfolded α-synuclein (SNAP) enrichment [11].

Another example is the focus on genetics in PD with the claim to identify PD-generating mechanisms as an initial step for disease modification or even cures [12]. As a result, antibody trials against proteins, i.e., on SNAP enrichment, were initiated. However, they largely failed and therefore did not provide any substantial therapeutic breakthroughs in terms of beneficial disease modification. Therefore, antibody SNAP therapies for PD with cinpanemab and prasinezumab, respectively, and with nilotinib as a compound for autophagic α-synclein degradation, have generated further doubts about pathologic protein enrichment as a cause for the onset and progression of PD [11,13,14]. Similar results will probably be found with therapeutic approaches in LRRK2 or GBA mutation carriers, i.e., with ambroxol [15]. As examples, the design of the performed trials suffers from short study intervals, missing considerations of co-morbid disorders and varying responses to dopamine-substituting drugs with their impact on applied rating scales for the assessment of disease progression [11,16]. Thus, the future stratification of therapies according to genetic disease mechanisms will probably fail again in PD simply due to the fact that genetically determined mitochondrial or lysosomal dysfunction is also influenced by epigenetic or environmental effects.

A way out of this dilemma is a better association between basic and clinical research in PD. The diversity between PD concepts of experimental and clinical insights has increased in recent years. Therefore, this Special Issue focusses on papers that provide a closer relationship between basic and clinical research in PD.

The proof-of-concept study by Hegelmaier and co-workers on a dietary intervention alone or with the additional of physical colon cleaning demonstrated that gut microbiome changes may result from vegetarian diet and fecal enema and persist during a one-year follow-up investigation. Interestingly, the motor behaviour in PD patients also improved. Therefore, the authors suggest dietary intervention and bowel cleansing as an additional non-pharmacologic therapeutic option for PD patients [17]. This paper is an essential contribution to the emerging discussion on the impact of the microbiome on the pathogenesis of Parkinson’s disease. It remains to be determined whether these various gut flora changes are additionally influenced by chronic PD drug treatment, i.e., with levodopa, as an epigenetic drug effect, or whether they result from weight reduction, which may contribute to a better response to levodopa [18,19,20,21,22].

There is an ongoing discussion about the value of SNAP determinations as a laboratory diagnostic tool for the diagnosis and the further progression of PD. The paper by Barkovits et al. pointed out that blood contamination in cerebrospinal fluid may increase the sensibility for detection of SNAP. Thus, this paper highlights the difficulty of an exact and authentic quantification of the SNAP protein in cerebrospinal fluid. With circumstantial evidence, these results are in line with the classification of SNAP as a more unspecific, less reliable biomarker for PD and emphasize the importance of clean cerebrospinal fluid collection in clinical practice [23].

Another diagnostic tool for PD and related disorders is transcranial sonography, which is an easy to perform procedure in clinical practice for the evaluation of echogenic basal ganglia alterations in patients with extrapyramidal movement disorders. Trials with small sample sizes reported a more frequent hyperechogenic nucleus lentiformis in atypical PD-like syndromes such as multiple system atrophy with predominant Parkinsonian features or the progressive supranuclear palsy. The meta-analysis performed by Richter et al. confirmed the higher prevalence of nucleus lentiformis hyperechogenicity. They suggest a histopathological work up for possible causes of this phenomenon, which may also result from the metal ion accumulation in PD and related disorders [24]. However, the value of transcranial sonography for the visualization of this metal ion enrichment as a result of dopamine oxidation is under debate [25,26].

Therapeutic oxidative stress decline is in the focus of the experimental work by Zhou et al., who convincingly demonstrate the beneficial protective effects of tea phenols in various cell death models, all of which are discussed in the context with PD [27]. This ground-breaking experimental paper provides a further milestone in the ongoing discussion of nutritional supplementation of green tea for neuroprotection in PD, which still lacks a convincing confirmative long-term clinical trial in PD patients to date [28,29,30,31].

The work by Leupold et al. investigated PD patients and controls within a melanin and neuromelanin fluorescence trial. It discussed the association between the onset of PD and of melanoma as a mirror of configuring change in neuromelanin or skin melanin [32]. This study found no further specific indications for an association between PD and melanoma pathogenesis onset, despite an ongoing discussion, in terms of exposure to certain PD drugs [33,34].

Fibroblasts from a young female PD patient and her relatives were investigated to demonstrate that dysfunction of the endocytic or autophagic lysosomal pathway is associated with mitochondrial impairment. Synergistic alterations in lysosomal functions and mitochondrial biogenesis were shown. Therefore, it appears more likely that they derive from a mitochondrial genetic defect, which blocks mitochondrial turnover and leads to premature cellular senescence in PARK2-PD fibroblasts. It still remains to be elucidated whether these changes represent potential mechanisms that may contribute to the loss of dopaminergic neurons [35].

Gait disturbances are the focus of the investigation performed by Janeh et al. [36]. They show that support by virtual manipulation techniques may help to ameliorate the still largely unexplained gait disturbances, such as the freezing of gait phenomenon, in PD patients. These short-term, positive outcomes are at least partially in line with further therapeutic approaches in PD patient rehabilitation facilitated by visual, acoustic stimuli or augmented attention by physiotherapists with the osteopathy technique [37,38,39,40,41]. Gait problems in PD probably also mirror the dysfunction of crosstalk within the basal ganglia network.

The in-depth review by Mallet et al. summarizes the current knowledge on basal ganglia function in relation to motor programming and execution, procedural learning, and cognitive and emotional behaviour. In PD, the basal ganglia interplay is primarily affected by the degeneration of midbrain dopaminergic neurons localized in the substantia nigra pars compacta. In this review, the authors also extensively discuss the impact of the functional relevance of dopamine modulation outside the striatum in both normal and pathological conditions, such as PD [38].

In summary, this Special Issue reflects the close relationship between experimental and clinical research in PD and underlines the necessity for further cooperation between experimental and clinical research in PD.
